# Supplementary material for: Facilitating Factors and Barriers in the Return to Work of Working Women Survivors of Breast Cancer: A Qualitative Study
Source: Cancers (Basel). 2023 Jan 31;15(3):874. doi: 10.3390/cancers15030874 (PMC9913437; doi:10.3390/cancers15030874)
Supplement: Supplementary file 1 [file cancers-15-00874-s001.zip › cancers-2163133-supplementary.pdf]

Supplementary Material

Table S1. Themes of breast cancer on working women and issues discussed of health care professionals.

Themes	Debated questions
Impact of the disease on employment	• Do you think that women diagnosed with breast cancer have a higher rate of unemployment than other patients diagnosed with other pathologies (both cancer and other diseases)?<br>• What do you think are the factors that prevent women with breast cancer from returning to work?<br>• Do you think there are certain factors that make it easier for these women to return to work?<br>• What would you say they are?
Impact of clinical features and therapies applied on return to work	• Would you say that the stage at the time of diagnosis has any influence on the return to work of these patients? And the treatment that is subsequently carried out? And why?<br>• Do you think that treatment aimed at minimizing the accompanying symptoms of breast cancer (e.g. fatigue or asthenia) could facilitate these women's return to work?<br>• Regarding the physical and psychological sequelae of the cancer itself and its treatment, do you think they affect women's return to work?
Impact of coping by health care workers	• What medical actions do you think could be carried out to promote those facilitating factors that have been mentioned? And, on the contrary, do you have any idea of how to avoid those factors that are detrimental to reinsertion into the labor market?<br>• Do the specialists of your own specialty believe that they have been specifically prepared to collaborate in the return to work of their patients within the framework of their clinical/healthcare activity?<br>• Do they think that their support and follow-up and good understanding with the specialist can be an important factor in the patient's return to work?<br>• Do you think that they need to be sensitized and/or trained in this respect?<br>• Concerning the collaboration between occupational medicine specialists, hospital specialists who diagnose, treat, and rehabilitate patients and family doctors, do you think that there is a quality collaboration between all of them?<br>• In case there is not a good quality: How do you think it could be improved?

Table S2. Synthesis of opinions and perceptions in the focus group of health care professionals.

Diagnosis of illness	Perceived barriers to return to work	Proposals to facilitate the return to work
The specialists involved in the diagnosis and treatment of patients in the hospital center do not usually pay specific attention to aspects related to the patient's return to work.	Lack of training and awareness (of the professionals attending the patient from the moment of diagnosis) on the importance of the return to work for the health and quality of life of the patients and their possible role in this aspect.	Training and sensitization of physicians on the importance of providing basic support/accompaniment in the return to work.
Illness and unemployment are frequently associated with breast cancer diagnosis.	The type of activity performed can act as a barrier, with more physically demanding and lower skilled jobs often being the most difficult to return to.<br>Likewise:<br>- Diagnosis in advanced stages of cancer<br>- The most aggressive treatments<br>- The patient's level of education	To know what "providing support or accompaniment" by clinical-assistance medical specialties, other than Occupational Medicine, consists of.
Fear of not being able to go back to work/losing the job/not measuring up	Very important: emotional state and psychological consequences of the disease and fear of stigmatization at work.	Personalized attention in each case
Concern about sick leave	Limited access to the information/clinical data	Personalized attention<br>Motivation<br>Promote job adaptation

And for a return that is truly progressive and allows for the necessary job adaptation.	necessary for job adaptation by occupational physicians.	
	Legal vacuum, since it is not strictly regulated that breast cancer patients can have access to reduced working hours due to illness.<br>Nor is there any regulation regarding the gradual return to work, which in many cases the company cannot cope with.	Promote multidisciplinary collaboration between the different specialists involved in the care of breast cancer patients and specialists in Occupational Medicine.<br>Establish channels of cooperation and information flow to facilitate the support work of health professionals.
		Need for regulatory development to promote interdisciplinary collaboration among physicians and the progressive incorporation of women cancer survivors in their return to work.

Table S3. Themes of breast cancer on working women and issues discussed in the focus group session.

Clinical stages (themes)	Issues discussed
Situation at diagnosis	What were your first feelings upon learning of the diagnosis?<br>How did you think it would impact your daily life?<br>In your workplace: Did you inform your superiors of the diagnosis? And your colleagues?<br>If so, how and when?<br>If not: Why not?<br>What response did you get?

	During the assessment by the medical staff of the Breast Unit, were you asked about your work situation?<br>When did you start your sick leave?<br>Did you have any difficulties in accessing it?
Situation during treatment	How did you feel about not being able to practice your profession during this time?<br>At any time was the option of dismissal considered?<br>Have you maintained contact with co-workers during treatment (including superiors or human resources personnel)?<br>If so, do you think their support may have encouraged you to return to work?
Situation at the end of treatment	Regarding the physical and psychological sequelae derived from the cancer itself and its treatment, do you consider that they may have affected the performance of your job?<br>Have you been able to return to your job?<br>If so, under what conditions?<br>Under what conditions? Has your job been adapted?<br>How do you consider the treatment received by your bosses and colleagues?<br>How do you consider the treatment received by the hospital health professionals (kindness, availability, resolution of doubts)?<br>Were they able to resolve your doubts regarding your return to work?<br>What do you think what could be improved during each phase of the process to facilitate your return to work?

Table S4. Synthesis of opinions and perceptions in the focus group of women workers with breast cancer.

Related to the diagnosis of the disease	Perceived barriers to return to work	Proposals to facilitate the return to work
State of shock and vital uncertainty after diagnosis.	Uncertainty about the continuity in the performance of their previous activities due to the after-effects of the treatment.	Protocol functional recovery through rehabilitation programmes.
Progressive improvement in the emotional management of the situation.	"Depersonalization" of the worker: they consider that the company is not willing to assume the indirect costs associated with the illness.	Greater accuracy in the waiting times for pending medical-surgical procedures for better planning of time off work.
Most of them shared the diagnosis with their colleagues and superiors.	Follow-up of the disease involves periods of absence from work.	Better knowledge about breast cancer in companies would facilitate support for affected workers.
The socio-occupational situation is not addressed in medical consultations.	Physical and cognitive symptoms resulting from treatment.	Help and support from co-workers and superiors could favor the return to work and recovery.
They perceive the care provided by health professionals during the diagnostic and therapeutic process as "very good".		Legal advice to guarantee patients' rights
Start of temporary incapacity: simultaneously with the start of treatment.		

Table S5. Themes and issues discussed in the focus group session of health professionals.

Actions at company level (themes)	Issues discussed
The employee's cancer diagnosis and its impact on the company.	What does "being a good boss or manager" mean to you as far as employees with cancer are concerned? (Possible decrease in productivity, attention to employees...).<br>How did the absence of the employee affect your organization (need for replacement, colleagues, clients, direct and indirect economic impact)?<br>Do you think that the size of the company is a determining factor?<br>In any case did you consider that it was necessary to dismiss the employee?<br>How did you know that your employee had cancer, and do you think it was difficult for her to communicate this information?<br>Could you identify the barriers and facilitating factors in a woman who has who have overcome breast cancer?<br>During the illness, were you in contact with the employee?<br>What advice would you give to employers with cancer-stricken employees in relation to the experience (comment on both successes and failures, invite creativity, lessons learned)?
Tools to improve the process of reintegration into the labor market.	Do protocols and interventions exist in your companies that allow for the reintegration of female workers? (If they exist, explain how they take place and the role of the different actors: HR, Trade Unions, etc.).<br>Do you consider it necessary to adapt the workplace when the employee returns (length of the working day, type of work performed, etc.)?

	Do you consider that you have sufficient information related to the needs of an employee with breast cancer (follow-up, after-effects, job adaptation, etc.)?<br>Do you think that early cooperation with occupational physicians and HR professionals would facilitate a successful reintegration process?<br>What kind of resources (informative, educational, consulting, financial) would you like to have or do you think other employers would need in order to find the most appropriate solution?

Table S6. Opinions and perceptions expressed by company managers in relation to the moment of knowledge of the diagnosis of breast cancer, perceived barriers and facilitating factors.

Related to the diagnosis of the disease	Perceived barriers to return to work	Proposals to facilitate return to work
Emotional impact on the employee	Psychological impact after diagnosis	"Humanize the company". Take into account the needs of each employee from diagnosis to return to work and provide support during the process as far as possible.
Difficult communication of the diagnosis of the disease<br>.	High level of self-demanding after overcoming the disease.	Facilitate communication and understanding of the situation, and personalized support.<br>Encourage gradual reintegration and adaptation of the activity with the support of specialists in occupational medicine
Individualize the situation and needs of each case.	Lack of psychological support in the business environment	Need for psychologists and physicians

Increased awareness of the disease among colleagues and superiors.	Greater difficulty in taking sick leave in small companies.	Elaboration of protocols for companies/guides for return to work in women with breast cancer and the need for collaboration between specialists in Occupational Medicine and Family Medicine.
HR patient follow-up<br>From an administrative approach to one focused on workers' well-being	Lack of knowledge and confusion regarding the terms “disability” and “incapacity”.<br>In case of disabilities resulting from the process: possible benefits and tax advantages.	Creation of a legal framework that supports the worker and offers companies the necessary institutional support to deal with these situations.
	Lack of legal support for companies<br>Lack of contact between the specialists in charge of the patient's clinical care and cancer follow-up and the occupational physicians.	Reinforce multidisciplinary collaboration between: professionals who carry out hospital and primary care follow-up, occupational physicians, and HR professionals.
	Lack of coordination between primary care and mutual insurance companies.	
